# Comparative Toxicity of Size-Fractionated Airborne Particulate Matter Collected at Different Distances from an Urban Highway

**DOI:** 10.1289/ehp.0900730

**Published:** 2009-06-29

**Authors:** Seung-Hyun Cho, Haiyan Tong, John K. McGee, Richard W. Baldauf, Q. Todd Krantz, M. Ian Gilmour

**Affiliations:** 1 National Health and Environmental Effects Research Laboratory and; 2 National Risk Management Research Laboratory, U.S. Environmental Protection Agency, Research Triangle Park, North Carolina, USA; 3 Research Participation Program, Oak Ridge Institute for Science and Education, Oak Ridge, Tennessee, USA; 4 Office of Transportation and Air Quality, U.S. Environmental Protection Agency, Ann Arbor, Michigan, USA

**Keywords:** cardiopulmonary, chemical composition, inflammation, ischemia–reperfusion injury, mice, motor vehicle emissions, near road, particulate matter, size fraction

## Abstract

**Background:**

Epidemiologic studies have reported an association between proximity to highway traffic and increased cardiopulmonary illnesses.

**Objectives:**

We investigated the effect of size-fractionated particulate matter (PM), obtained at different distances from a highway, on acute cardiopulmonary toxicity in mice.

**Methods:**

We collected PM for 2 weeks in July–August 2006 using a three-stage (ultrafine, < 0.1 μm; fine, 0.1–2.5 μm; coarse, 2.5–10 μm) high-volume impactor at distances of 20 m [near road (NR)] and 275 m [far road (FR)] from an interstate highway in Raleigh, North Carolina. Samples were extracted in methanol, dried, diluted in saline, and then analyzed for chemical constituents. Female CD-1 mice received either 25 or 100 μg of each size fraction via oropharyngeal aspiration. At 4 and 18 hr postexposure, mice were assessed for pulmonary responsiveness to inhaled methacholine, biomarkers of lung injury and inflammation; *ex vivo* cardiac pathophysiology was assessed at 18 hr only.

**Results:**

Overall chemical composition between NR and FR PM was similar, although NR samples comprised larger amounts of PM, endotoxin, and certain metals than did the FR samples. Each PM size fraction showed differences in ratios of major chemical classes. Both NR and FR coarse PM produced significant pulmonary inflammation irrespective of distance, whereas both NR and FR ultrafine PM induced cardiac ischemia–reperfusion injury.

**Conclusions:**

On a comparative mass basis, the coarse and ultrafine PM affected the lung and heart, respectively. We observed no significant differences in the overall toxicity end points and chemical makeup between the NR and FR PM. The results suggest that PM of different size-specific chemistry might be associated with different toxicologic mechanisms in cardiac and pulmonary tissues.

During the last several decades, industrialization and urbanization have resulted in dramatic increases in vehicle-associated emissions. More than 50% of the total particulate matter (PM) emissions in urban areas are related to road traffic (Briggs et al. 1997). A number of studies have reported significant associations between traffic density or proximity to roads and various respiratory symptoms in children (Brunekreef et al. 1997; Edwards et al. 1994; Venn et al. 2001). A recent study from London reported that asthmatic individuals showed increased symptoms (lung dysfunction and inflammation) while walking around heavily trafficked Oxford Street compared with performing similar activities in Hyde Park (McCreanor et al. 2007). In addition, increased cardiovascular effects have also been reported with environmental exposure to vehicular-traffic–derived pollutants (Dockery et al. 2005; Peters et al. 2004).

Roadside aerosols are a complex mixture of different size particles that segregate into three fractions. Coarse PM [2.5–10 μm in aero-dynamic diameter (PM_2.5–10_)] is produced by abrasion of automobile brakes and tires and dispersion of road dust, whereas fine (PM_2.5_) and ultrafine (PM_0.1_) materials are emitted directly from the tail pipe or form as secondary aerosols through atmospheric reactions. Vehicle emission factors have been estimated from dynamometer emission monitoring, as well as from locational studies of tunnels and near-roadside environments (Kuhn et al. 2005; Phuleria et al. 2006; Riddle et al. 2008). These studies have reported larger PM emissions from diesel trucks compared with gasoline vehicles, with higher levels of direct vehicle emissions related to proximity to the roadside. Because the physicochemical characteristics of roadside airborne particles are also influenced by factors such as location, climatic conditions, traffic volumes, type of fuel and engine, and operating conditions, the toxic potential of these mixtures can be quite diverse (DeMarini et al. 2004; Gilmour et al. 2007; Kleinman et al. 2007; Seagrave et al. 2002; Singh et al. 2004). Direct exposure to combustion emissions has resulted in a wide variety of cardiopulmonary effects in animals and human volunteers (reviewed by Hesterberg et al. 2009; Lewtas 2007; Sydbom et al. 2001). Furthermore, many of these pathophysiologic changes have also been noted after inhalation exposure to ambient PM (Brook et al. 2002; Gong et al. 2003; Gurgueira et al. 2002; Samet et al. 2009; Sun et al. 2005), sometimes in association with traffic-related pollution. We designed the present study to characterize the chemical composition of size-fractionated airborne PM collected at either 20 or 275 m from an interstate highway and to determine the cardiopulmonary toxicity of the particles after oropharyngeal aspiration in mice.

## Materials and Methods

### Sample collection

We collected two consecutive weekly samples of airborne PM in three different size ranges (coarse, 2.5–10 μm; fine, 0.1–2.5 μm; ultrafine, < 0.1 μm) using ChemVol high-volume cascade impactors (model 2400, Rupprecht & Patashnick Co. Inc., Albany, NY) during the weeks of 26–31 July and 3–10 August 2006. Sampling sites were located approximately 20 m and 275 m from the nearest travel lane of a highway in Raleigh, North Carolina, and are referred to as the near road (NR) and far road (FR) sites, respectively. The sites were chosen based on prevalent wind direction and distance from the highway and represented potentially high and low exposure to pollution emitted by traffic on the adjacent highway (Kuhn et al. 2005; Riddle et al. 2008). The vehicle fleet mix at the highway consisted mostly of light-duty, gasoline-powered motor vehicles. Detailed description of sampling site characteristics have been published previously (Baldauf et al. 2008). The coarse and fine fractions of PM and the ultrafine PM were collected onto precleaned, preweighed polyurethane foam (PUF) and polypropylene fiber filters, respectively. The amount of material collected was determined gravimetrically. The PM was extracted for chemical analyses and toxicity testing.

### PM extraction and recovery

For the coarse and fine PM fractions, PUF strips were transferred to sterile 50-cc polypropylene tubes and submerged in 35 mL of analytical-grade methanol. The tubes were placed in an ultrasonic water bath at < 25°C for 1 hr at 100% power, 25 kHz frequency, and normal ultrasonic mode (model TI-H15, Elma Hans Schmidbauer GmbH & Co. KG, Singen, Germany). After sonication, a sterile Teflon rod was pressed against the PUF to help liberate material in the pores. Extracted material was decanted into preweighed sterile polypropylene 50-cc tubes. For the ultrafine PM fractions, a polypropylene fiber filter was placed in a sterile 4-L glass beaker with the PM-collected side downward and submerged in 150 mL methanol. The beaker was covered with Parafilm and sonicated for 1 hr under the same conditions as the PUF filters. The extracted material was decanted into a sterile 250-mL polypropylene bottle. The beaker was rinsed with additional volumes of methanol and then combined with extracted material. Aliquots of all size fractions for each collection week were evaporated to dryness under nitrogen, and the tubes were reweighed. PM recovery efficiency was calculated as the difference between the pre- and postweight of the tubes, divided by the PM mass collected on the weekly filters.

After determination of PM mass, the dried materials of each sample were dissolved in 100% methanol, followed by addition of 0.9% sterile saline, producing a PM suspension containing 0.5% methanol and 10 mg/mL PM. A control saline solution contained 0.5% methanol. The reconstituted PM suspensions and saline were sonicated, vortexed for 1 min, and analyzed for chemical constituents; 1-mL aliquots of the material were stored at −80°C until toxicity testing. For the toxicologic study, we combined two weekly PM suspensions of the same size fraction and location to produce the same mass ratio of collected PM samples from the individual weeks.

### Chemical analyses of PM

We analyzed NR and FR PM samples of each week for inorganic elements, ions, and carbon content. For elemental analysis, 2-mg aliquots of PM suspension in saline used for the animal exposures were digested in 10% nitric acid to solubilize the metals (Wichers et al. 2006). Supernatants separated by centrifugation were diluted to a final concentration of 1.5% HNO_3_ and then assayed for 28 inorganic elements by inductively coupled plasma-optical emission spectrometry (ICP-OES) using U.S. Environmental Protection Agency (EPA) Method 200.7, rev4.4 (U.S. EPA 1994). For ionic components, 250-μg aliquots of the saline PM suspensions were diluted with 6 mL ultrapure water and analyzed for nitrate, sulfate (SO_4_^2−^), chloride, sodium, ammonium (NH_4_^+^), potassium, magnesium, and calcium by ion chromatography using EPA Compendium Method IO-4.2 (U.S. EPA 1999) with calibration and quality control standards obtained from Dionex Corporation (Sunnyvale, CA) and Alltech Associates, Inc. (Deerfield, IL). Excessive amounts of sodium and chloride were present due to the saline in the PM suspension and were not reported in the result (Table 1). For carbon analysis, 1-mg aliquots of PM in methanol were pipetted onto prefired 1.45-cm^2^ quartz filters, dried, and analyzed by thermo-optical methods based on sequential pyrolytic vaporization and detection by transmittance using a carbon analyzer (model 107A; Sunset Laboratory Inc., Tigard, OR) (Birch and Cary 1996).

### Endotoxin measurements

Particle suspensions in saline were sonicated, vortexed for 15 min, and centrifuged for 15 min at 2,100 rpm. Supernatants were collected and diluted in endotoxin-free water at PM-equivalent concentrations of 1, 0.1, and 0.01 mg/mL. Endotoxin measurements were performed using the *Limulus* amoebocyte lysate assay (QCL-1000; Cambrex BioScience Inc., Walkersville, MD).

### In vivo *toxicity of PM.* Experimental animals

Female CD-1 mice were obtained from Charles River Laboratory (Raleigh, NC) and housed five per cage in a barrier-isolated animal facility approved by the Association for Assessment and Accreditation of Laboratory Animal Care. Food and water were provided *ad libitum*. We used two different groups of mice: one for pulmonary assessment and blood and tissue collection [*n* = 7/group (total, *n* = 196); 7–8 weeks of age; body weight, 29.7 ± 2.3 g], and another for cardiac evaluation [*n* = 5/group (total, *n* = 40); 16 weeks of age; 33.8 ± 0.8 g]. All animals were treated humanely and with regard for alleviation of suffering in accordance with National Institutes of Health guidelines (Institute of Laboratory Animal Resources 1996).

### Pharyngeal aspiration

Surgical anesthesia was induced using a commercially available system (VetEquip Inc., Pleasanton, CA) to generate a 5% isoflurane/oxygen mixture. PM suspensions (25 or 100 μg) or the saline vehicle were administered by pharyngeal aspiration (Gilmour et al. 2007). Similarly, a separate group of mice received 2 μg lipopolysaccharide (LPS) (*Escherichia coli* endotoxin; 011:B4 containing 10^6^ unit/mg material; Sigma, St. Louis, MO) as a positive control to demonstrate maximal inflammatory response to this well-characterized inflammatory agent.

### Pulmonary responsiveness testing

Two hours before euthanasia and necropsy, a subgroup of mice was challenged with increasing concentrations of methacholine (MCh) aerosol in order to determine pulmonary responsiveness. We used a 12-chamber whole-body plethysmograph system using Buxco BioSystem XA software (Buxco Electronics, Wilmington, NC) to measure ventilatory parameters and the enhanced pause (Penh), an index of airflow limitation and a surrogate for bronchoconstriction (Hamelmann et al. 1997). After measuring baseline parameters for 10 min, an aerosol of saline or 6.25, 12.5, or 25 mg MCh/mL saline was pulled through each plethysmograph at a constant and identical flow rate (2 mL/sec). Response to saline and MCh was measured for 10 min, both during the 2-min aerosolization period and then for 8 min afterward. A MCh dose–response curve was then constructed for each animal. In addition, by interpolating the dose–response curve, the concentration of MCh required to produce a doubling of the Penh from the saline (EC_200_) was calculated for each animal.

### Biological sample collection and hematology

At 4 or 18 hr postaspiration, mice from each treatment group were euthanized and plasma was obtained by cardiac puncture using a 1-mL syringe containing 50 μL EDTA acid as anticoagulant. The trachea was cannulated and the left main stem bronchus occluded while the right lung lobes were lavaged once with 3 vol of 0.6 mL (35 mL/kg) Hank’s balanced salt solution (HBSS) maintained at 37°C. The recovered bronchoalveolar lavage fluid (BALF) was centrifuged at 130 × *g* for 10 min at 4°C with an aliquot stored at either 4°C for biochemical measurement or −80°C for cytokine measurement. Cell pellets were resuspended in 1 mL HBSS. Total BALF cell count for each mouse was measured with a Coulter Counter (Beckman, CA). Samples (200 μL) were prepared in duplicate onto slides using a Cytospin (Shandon, PA) and stained with HEMA 3 stain set (Fisher Diagnostics, Middletown, VA) for cell differentiation determination using light microscopy. Hematologic data including total white blood cells, total red blood cells, hemoglobin, hematocrit, mean corpuscular volume, mean corpuscular hemoglobin concentration, platelets, and lymphocytes were measured using a Coulter AcT 10 Hematology Analyzer (Beckman Coulter Inc., Miami, FL). Residual blood samples were centrifuged and processed to plasma for subsequent clinical chemistry analyses.

### Cytokine measurements

Concentrations of the proinflammatory cytokines macrophage inhibitory protein-2 (MIP-2), tumor necrosis factor-α (TNF-α), and interleukin-6 (IL-6) in BALF were measured by enzyme-linked immunosorbent assay using mouse Quantikine kits (R&D Systems, Minneapolis, MN). The lowest value in the standard curve for each cytokine was 15.625, 19.531, and 15.625 pg/mL for MIP-2, TNF-α, and IL-6, respectively, and all values below these lowest values were replaced with a fixed value of one-half of the lowest value.

### BALF and plasma biochemistry

Lactate dehydrogenase (LDH), microalbumin, *N*-acetyl-β-D-glucoaminidase, and total protein were determined in BALF. Creatine kinase, amino-*S*-transferase (AST), alanine transaminase (ALT), LDH, α1 antitrypsin (A1AT), haptoglobin (HPT), C-reactive protein (CRP), and fibrinogen (FIB) were analyzed in plasma. All biochemical assays were modified for a Konelab 30 clinical chemistry analyzer (Thermo Clinical Labsystems, Espoo, Finland), as previously described (Gilmour et al. 2007).

### Cardiac perfusion

We used a separate group of mice for assessment of cardiac toxicity. Mice aspirated 100 μg each of coarse, fine, or ultrafine PM collected from both NR and FR collection sites. At 18 hr postaspiration of PM, *ex vivo* Langendorff cardiac perfusion and subsequent ischemia reperfusion were performed as described previously (Tong et al. 2009). The Langendorff perfusion model has been used to investigate cardiac responses after PM exposure in experimental animal studies (Bagate et al. 2006; Wold et al. 2006). We used the PowerLab system (AD Instruments, Milford, MA) to collect and process the left ventricular developed pressure (LVDP), heart rate, maximum of the first derivative of left ventricular pressure rise (+dP/d*t*_max_), and minimum of the first derivative of left ventricular pressure fall (−dP/d*t*_min_) data. One heart did not achieve a developed pressure of at least 90 cm H_2_O and was excluded from data analysis. Coronary flow rates were measured before and after induced ischemia reperfusion. After ischemia reperfusion, recovery of cardiac function and infarct size were measured at 1 and 2 hr of reperfusion, respectively. A detailed procedure of necrosis evaluation has been described by Tong et al. (2009).

### Statistical analysis

We analyzed data in three increments. Initially, we performed a two-way analysis of variance (ANOVA) to evaluate BALF and plasma data, and we used a three-way ANOVA to evaluate the Penh data. These models examined overall effects of particle size (coarse, fine, and ultrafine) and sampling location (NR and FR sites) in the two-way ANOVA, or the influence of particle size, sampling location, and MCh concentration [levels: −1 (baseline), 0 (saline), 6.25 (6.25 mg MCh/mL saline), 12.5 (12.5 mg MCh/mL saline), and 25 (25 mg MCh/mL saline)] in the three-way ANOVA. Control exposure groups (saline and LPS) were not included in this analysis. The second step employed a one-way ANOVA model using an independent variable formed by combining location and size into a single treatment group parameter, and the saline control was included in the model. In the third step we used Dunnett’s test to compare the effect between each PM-exposed group and saline control group, adjusting significance level for multiple comparison. All the data were analyzed separately by each postexposure time period and PM dose. Significance was assigned at *p* < 0.05.

## Results

### Particle characteristics

Table 1 shows the collected particle masses and recovered fractions after extraction from two weekly samples from each sampling location, as well as the chemical analyses. Approximately 20% more total PM mass was collected at the NR site than at the FR site. Coarse and fine NR PM masses were 27% and 20% greater, respectively, than corresponding fractions of FR PM; we found no difference in ultrafine fractions. The percent recovery ranges of size-fractionated PM after methanol extraction and evaporation were 54–80%, 41–70%, and 68–101% for the coarse, fine, and ultrafine PM samples, respectively. The percent recoveries were relatively higher for FR PM and showed greater consistency between weekly samples compared with NR PM. Losses likely occurred from particles remaining in the filter substrates as well as during the extraction procedure.

Calcium (Ca^2+^), potassium (K^+^), and nitrate (NO_3_^−^) were much enriched in the coarse fractions, whereas SO_4_^2−^ and NH_4_^+^ were more abundant in the fine and ultra-fine fractions. The ion chromatography analyses of calcium and SO_4_^2−^ contents showed good agreement with elemental analysis using ICP-OES. Calcium levels were 1.6 times higher in the ultrafine fraction of NR PM compared with the FR sample. The total carbon consisted of 19–22% of total mass of coarse and fine PM and 24–26% of ultrafine PM without apparent difference by location. Organic carbon amount ranged between 19% and 26%, with similar levels at both locations. Elemental carbon content was therefore much lower than the organic carbon and was mostly enriched in the fine fractions. The sum of elemental carbon in all fractions of NR PM was 2.4 times higher than that of FR PM. With regard to biogenic components, the coarse PM contained approximately 100 times higher levels of water-soluble endotoxin than did the fine and ultrafine PM, and the NR coarse PM had 2 times more measureable endotoxin than did the FR coarse PM.

Table 2 shows the inorganic elemental constituents of each size fraction from both locations. The concentrations of elements contained in NR PM [aluminum (Al), barium (Ba), calcium (Ca), cadmium (Cd), chromium (Cr), copper (Cu), iron (Fe), manganese (Mn), lead (Pb), antimony (Sb), silicon (Si), strontium (Sr), titanium (Ti), vanadium (V), zinc (Zn)] were at least 30% higher per gram of PM mass in any of three size fractions than in the FR PM. Lead (Pb) and tin (Sn) were present in relatively higher concentrations in the FR fine and ultrafine samples than in the NR samples.

**Figure 1:**
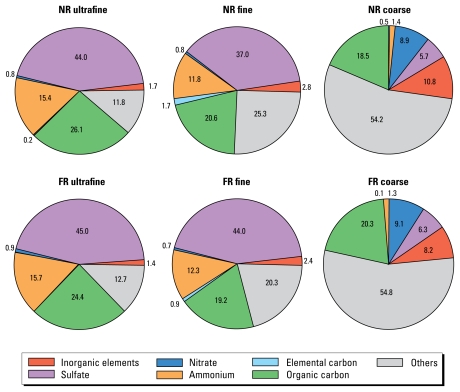
Mass balances (%) of major classes of chemical components in each fraction of NR and FR PM in the saline suspension used for pharyngeal aspiration on mice.

Figure 1 shows the mass balance of major classes of chemical components from each saline-suspended PM sample used for pharyngeal aspiration in mice. Overall, the chemical profile of the coarse fraction had a pattern different than that of the fine and ultrafine fractions, whose relative proportions of the major chemical classes were quite similar. Furthermore, none of the ratios of these major classes was appreciably altered by location. Unidentified moieties increased with particle size: 12–13% for ultrafine PM, 20–25% for fine PM, and 54–55% for coarse PM. These were presumably mineral components such as refractory aluminosilicates and associated alkali and oxygen components, which, unlike the anthropogenic metals, did not fully dissolve in nitric acid.

### Inflammatory biomarkers in BALF

**Figure 2:**
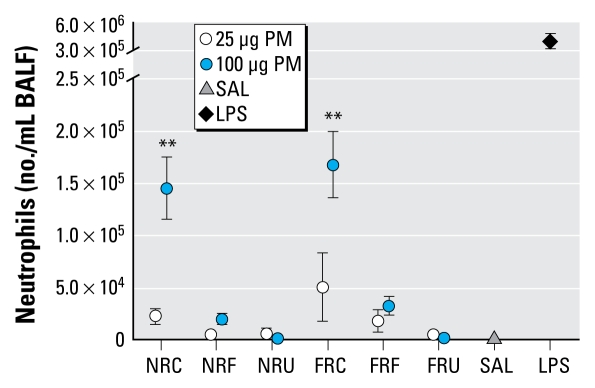
Number of neutrophils in BALF 18 hr postexposure to NR and FR PM (*n* = 7 mice/group). Abbreviations: C, coarse; F, fine; SAL, saline; U, ultrafine. Error bars indicate SE. **p < 0.01 compared with saline.

With the exception of cytokines, which peaked at the 4-hr time point, all other markers of injury and inflammation were highest at 18 hr postaspiration. Saline aspiration produced no apparent increase in markers compared with historic untreated control data, whereas LPS-treated animals had high values of inflammation, cytokines, and markers of lung injury. The overall statistical analysis of numbers of polymorphonuclear neutrophils (PMNs) by two-way ANOVA showed a significant particle size effect but no sampling location effect. At 18 hr postexposure, for the 100-μg dose, the PMN numbers were significantly higher in the coarse PM-exposed groups compared with the saline group regardless of sampling location, comprising 34–39% of the total cells (Figure 2), whereas the 25-μg PM dose did not induce a significant effect. We observed no significant increases in PMNs for any of the fine or ultrafine PM at either concentration or location. Lymphocyte numbers were also significantly increased (< 1% of the total cells) at 18 hr postexposure to the FR coarse PM of 100 μg but not by the NR coarse PM (data not shown). We found no significant differences in numbers of alveolar macrophages between the various treatment groups, although we did detect particles in the alveolar macrophages from PM-exposed mice.

**Figure 3:**
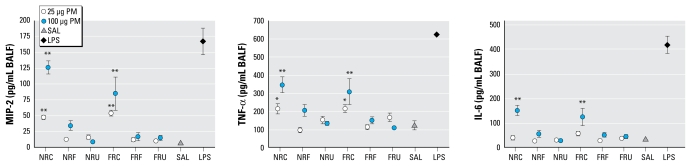
Proinflammatory cytokines of MIP-2, TNF-α, and IL-6 in BALF 4 hr postexposure to NR and FR PM (*n* = 7 mice/group). Abbreviations: C, coarse; F, fine; SAL, saline; U, ultrafine. Error bars indicate SE. *p < 0.05, and **p < 0.01 compared with saline.

Overall, the production of cytokines increased in a dose-dependent manner. The analysis of inflammatory cytokines by two-way ANOVA showed a particle size effect for MIP-2, TNF-α, and IL-6. At 4 hr (Figure 3), both the 100-μg and 25-μg doses of NR and FR coarse PM significantly increased the production of the MIP-2 and TNF-α. The responses to both NR and FR coarse PM were still significant with the 100-μg dose at 18 hr and to a lesser extent with the 25 μg (TNF-α only; data not shown). We found no significant differences for the other size fractions of any doses at any time points. At 4 hr (Figure 3), IL-6 was significantly increased with the high-dose coarse samples from both locations, and this effect persisted at 18 hr for the FR-sample (data not shown). LPS responses were much higher than PM at the 4 hr, indicating that the mice were capable of responding to an inflammatory agent.

**Figure 4:**
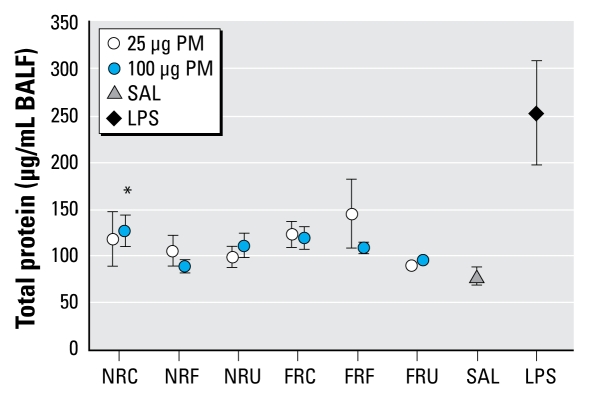
Total protein in BALF at 18 hr post-aspiration of both NR and FR PM (*n* = 7 mice/group). Abbreviations: C, coarse; F, fine; SAL, saline; U, ultrafine. Error bars indicate SE. *p < 0.05 compared with saline.

The overall statistical analysis of lung injury end points showed no significant effect by either particle size or sampling location. Analysis of individual treatment groups indicated that only total protein in BALF was increased at a small but significant degree with the 100-μg NR coarse PM at the 18 hr post-exposure (Figure 4). LPS treatment showed a clear increase in BALF protein concentrations over saline controls at 18 hr. For complete data for pulmonary markers and responsiveness to MCh see Supplemental Material, Table S1 (doi:10.1289/ehp.0900730.S1 via http://dx.doi.org/).

### Hematologic markers in whole blood and plasma

We monitored a variety of cellular and humoral markers in whole blood and plasma. The overall analysis by two-way ANOVA showed no significant effect by either particle size or location. In subsequent analyses, we found that the NR coarse PM sample caused a significant increase in creatine kinase in the plasma at 4 hr postexposure (data not shown), indicating systemic effects after aspiration with this material. We found no significant changes in cell numbers, platelets, or hematocrit markers or in plasma concentrations of A1AT, ALT, AST, CRP, FIB, HPT, or LDH.

### Pulmonary responsiveness testing

**Figure 5:**
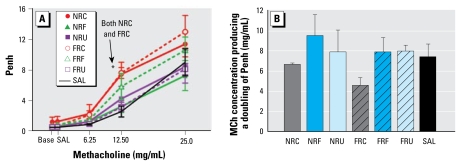
Changes in respiratory responsiveness after MCh inhalation, measured by whole-body plethysmographs 18 hr after aspiration of 100 μg PM (*n* = 7 mice/group). (*A*) Penh. (*B*) MCh concentration producing a doubling of the Penh compared with saline vehicle. Abbreviations: Base, baseline; C, coarse; F, fine; SAL, saline; U, ultrafine. Error bars indicate SE. *p < 0.05 compared with saline aspiration treatment.

The overall analysis of MCh responsiveness by three-way ANOVA indicated a significant effect by particle size and MCh dose. In the subsequent analyses, animals exposed to 100 μg of either NR or FR coarse PM showed significantly enhanced pulmonary responsiveness to the 12.5 mg/mL MCh dose at 18 hr postexposure compared with the saline group (Figure 5A). The 25-mg/mL MCh dose also enhanced pulmonary responsiveness but not to a significant level. When the data were transformed to reflect the EC_200_ (Figure 5B), we detected no statistical significance for the location and size factors. Nevertheless, mice exposed to 100 μg coarse PM required a non-significant but notably lower dose of MCh to achieve a 100% increase in Penh compared with the saline group.

### Cardiac ischemia–reperfusion injury

To examine the cardiac effects of the various PM samples, we isolated hearts at 18 hr after aspiration of 100 μg PM, and perfused and evaluated them for baseline hemodynamics before undergoing ischemia reperfusion. The baseline hemodynamics are shown in Table 3. The coronary flow rates in the NR fine and ultrafine groups were significantly lower than that of the saline group. We also found a notable but nonsignificant reduction in the coronary flow rate in the FR ultrafine group. We observed increases in baseline LVDP and +dP/d*t*_max_, and decreases in −dP/d*t*_min_ in the coarse and ultrafine groups of both NR and FR PM, but these were not significant.

**Figure 6:**
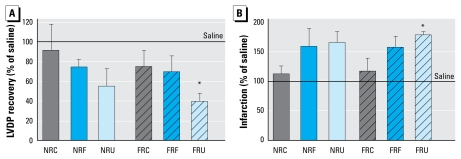
Post-ischemia–reperfusion cardiac end points in mice treated with 100 μg PM compared with saline control mice 18 hr postexposure (*n* = 5 mice/PM treatment group; *n* = 10 mice/saline group). (*A*) Recovery of LVDP. (*B*) Infarction. Abbreviations: C, coarse; F, fine; U, ultrafine. Error bars indicate SE. *p < 0.05 compared with saline.

After ischemia reperfusion, the FR ultra-fine group showed significantly lower recovery of postischemic LVDP (Figure 6A) and increased infarct size (Figure 6B) compared with the saline controls. For representative photomicrographs, see Supplemental Material, Figure S1 (doi:10.1289/ehp.0900730.S1). The NR ultrafine group also showed similar responses at a nonsignificant level. The coarse PM groups showed the smallest changes followed by fine PM groups, indicating these effects were dependent on particle size fraction. Overall, the results showed that ultrafine PM exposure increased ischemia–reperfusion injury in the mouse heart.

## Discussion

Many studies have compared the physico-chemical characteristics and relative toxicity of size-fractionated PM from different sources and locations. In general, coarse PM obtained from ambient air sampling contains larger amounts of bacterial endotoxin and insoluble crustal components than smaller size fractions of PM and, on a mass basis, have more pro-inflammatory potential than do fine or ultra-fine fractions (Becker et al. 2005; Dick et al. 2003; Gilmour et al. 2007; Happo et al. 2007). Combustion aerosols in the fine and ultra-fine modes can also generate significant lung inflammation (Gilmour et al. 2004; Seagrave et al. 2005; Singh et al. 2004), whereas simple chemicals such as carbon black and titanium dioxide tend to cause stronger effects as surface area increases and size decreases (reviewed by Donaldson et al. 1998). These results suggest that inflammatory potential is dependent on both chemistry and particle size, and that effects may vary depending on the method of collection, freshness of the sample, and coconstituents. Near roadway environments are of particular interest because they comprise an array of freshly generated and aged particles that coexist in each of the three broad size ranges. Although it has been known for some time that the size, number concentration, and chemistry of PM change with distance from highways (Kuhn et al. 2005), toxicity data for PM samples from different proximities to roadways have only recently been examined. Kleinman et al. (2007) recently reported that NR (50 m) emissions in the fine and ultrafine range significantly enhanced allergic responses in sensitized animals compared with exposures that occurred at FR locations (150 m). However, toxicity data simultaneously comparing cardiac and pulmonary effects after exposure to each of the size fractions from NR and FR locations require further investigation.

Traffic-associated airborne particles feature a trimodal size distribution typically found in other ambient samples and display a distinct chemical profile related to the vehicle fleet mix, operating conditions, meteorology, distance from highway, and presence of other air pollutants. In this study, NR PM was more enriched with inorganic elemental compounds than FR PM. Specifically, the coarse NR PM fraction contained more Ba, Cu, Sb, Zn, Fe, Pb, and Cd, which have been attributed to brake and tire wear (Querol et al. 2007; Wåhlin et al. 2006), and Al, Mn, and Si, associated with road dust, asphalt, and concrete. The fine and ultrafine fractions of NR PM contained larger amounts of Ba, Ca, Fe, Si, Sb, Ti, and V compared with FR PM. Along with some overlap with coarse PM, these elements are also components of lubricating and fuel oils (Dyke et al. 2007) and are emitted in vehicle exhaust (McDonald et al. 2004). The enrichment of these elements in NR PM indicated a significant contribution of traffic-related PM, whereas the high SO_4_^2−^ concentrations in the fine and ultrafine fractions at both locations likely reflected locally generated traffic emissions as well as the regional pollutant profile consistent with the chemical makeup of fine PM in the eastern United States (U.S. EPA 2008). Relatively small amounts of elemental carbon in NR PM reflected most of the vehicle fleet at the highway being gasoline-powered vehicles (Baldauf et al. 2008).

The toxicity results showed that inflammatory responses in mice were increased in a dose-dependent manner after aspiration of coarse PM samples, whereas treatment with equal masses of the smaller particles had much less effect. These findings are in agreement with previous reports from different cities across the United States and Europe that employed this sampling methodology and type of bioassay (Dick et al. 2003; Gilmour et al. 2007; Happo et al. 2007; Hetland et al. 2005). It should be noted that more coarse and fine PM collected at the NR location over the same sampling period would have resulted in a greater cumulative exposure compared with FR location, whereas the experimental exposure regimen described here was designed to compare toxicity on an equal mass basis. Furthermore, traffic-associated gases and volatile compounds that were not collected by the PM samplers are steadily diluted with distance from the roadway (Baldauf et al. 2008) and may be major contributors to reported health effects associated with proximity to roadways.

Much of the proinflammatory effect we observed from coarse PM was likely caused by the presence of LPS, whereas the smaller sized PM samples that produced no inflammation contained very little LPS. The NR coarse sample had twice as much LPS as did the FR course sample per gram of material but did not have substantially increased toxicity, suggesting that the effects were not solely driven by this substance. In addition to LPS, the coarse PM comprised many other soluble and insoluble components capable of activating lung cells and inducing chemotactic mediators with the subsequent recruitment of inflammatory cells (Hetland et al. 2004). Chemical and source apportionment analysis from the European study indicated that other components, including sea salt, soil-related elements, indicators of traffic, and nitrates, were also related to the inflammatory responses of coarse PM (Happo et al. 2007, 2008).

In agreement with the reports mentioned above, in the present study aspiration of fine or ultrafine PM caused little in the way of pulmonary inflammation despite the dose being as high as 100 μg per mouse. Because equivalent dosing of source-derived material, including fine and ultrafine coal fly ash, diesel exhaust particles, and oil fly ash (Gavett et al. 1999; Gilmour et al. 2004; Singh et al. 2004), produce varying degrees of pulmonary inflammation, this would suggest either that the fine and ultrafine ambient PM samples were less toxic than these source-specific PM samples, or that the atmospheric aging, collection and extraction procedures, or the presence of coconstituents reduced the activity of the sample.

In contrast to the pulmonary responses, the fine and ultrafine PM caused a decrease in coronary flow rate; after ischemia reperfusion, recovery of cardiac function was decreased; and the infarct size was increased by ultrafine PM. Previous studies have also reported decreases in vascular function and postischemic recovery with exposure to urban ambient ultrafine PM (Cozzi et al. 2006) and traffic-related ultrafine PM (Hwang et al. 2008; Peretz et al. 2008) in human volunteers and animals. Overall, the results suggest that ambient ultrafine PM has a higher potential to cause vascular dysfunction and cardiac damage than other size fractions and provides experimental support for recent epidemiologic and clinical reports (reviewed by Brook et al. 2004; Mills et al. 2009). There was also some suggestion that the NR ultrafine PM had greater effects on vascular function, but the FR ultrafine PM caused slightly more cardiac tissue damage after ischemia. Possibly these differences could be explained by the higher concentrations of elemental carbon at the NR location with increased amounts of heterogeneous organic aerosols (presumably secondary organic aerosols) at the distant location as has been previously reported (Kuhn et al. 2005; Riddle et al. 2008).

Although we did not observe cardiac effects for every particle size and location, the results illustrate that extrapulmonary responses can occur after fine or ultrafine PM exposure in the absence of detectable lung inflammation and injury. Several human exposure studies have shown that fine and ultrafine PM affects cardiovascular responses without significant effects on the respiratory tract (Gong et al. 2003; Samet et al. 2009). In one specific experimental example, inhalation of zinc sulfate did not cause lung injury in rats, but the soluble metal translocated to the cardiac tissue and inhibited phosphatases and kinase activation with subsequent effects on cardiac function (Wallenborn et al. 2008, 2009). It has also been demonstrated that insoluble ultrafine PM can translocate from the lungs to the circulation and be detected in other organs, including heart and liver (Furuyama et al. 2009; Takenaka et al. 2004). PM or its soluble constituents may be able to induce vasoconstriction by increasing endothelin release (Brook et al. 2002; Cozzi et al. 2006; Peretz et al. 2008). Reactive oxygen species (ROS), contained in the PM or endogenously generated in the lungs and heart, could also result in tissue damage (Gurgueira et al. 2002), which would decrease an ability to recover cardiac function and increase myocardial infarction after ischemia. Also ROS could affect the nitric oxide pathway and further contribute to the progression of atherosclerosis or other ROS (Sun et al. 2005).

On an equivalent mass basis, significant pulmonary and cardiac effects were observed with the coarse and ultrafine PM samples, respectively; however, in reality, unequal mass distributions for the different size fractions would likely affect the extent of toxicity. Particle penetration and deposition efficiency after aspirations exposure would also not reflect site-specific tissue doses after inhalation of heterogeneous aerosols (Foster et al. 2001). These challenges can be overcome in toxicologic and clinical studies by developing advanced exposure techniques to separate, characterize, and deliver different-sized particles and accompanying gases from complex air pollution atmospheres in order to improve understanding of the physicochemical basis of air pollution health effects.

## Conclusions

On a comparative mass basis, mice exposed to coarse PM from sites proximal to a highway showed significant increases in neutrophils, IL-6, MIP-2, TNF-α, and protein in BALF and altered pulmonary function in healthy mice with no noticeable effects on the heart. Meanwhile, the same dose of ultrafine PM affected the cardiac system in the absence of any pulmonary responses. Chemical analyses showed clear compositional difference between coarse PM and smaller PM but less overall changes in the mass balance between NR and FR locations and little in the way of altered biological effects. However, the higher levels of coarse and fine PM (as well as gases and vapors) next to the highway would presumably have resulted in increased exposure and potential for more severe health effects. Further studies are needed to examine real-time toxicity of NR and FR atmospheres to identify the levels and types of particle and gas phase components that cause health effects in multiple organ systems. This type of work will ultimately expand knowledge on the chemical composition and health effects of air pollution mixtures between NR and FR sites and could be used to improve human health risk assessment and provide guidance for planning of new roads and communities.

## Figures and Tables

**Table 1:** Collected mass and extraction recovery and chemical compositions in PM fractions from NR and FR sites, and endotoxin content in particle extract of PM fractions.

	NR			FR		
	Coarse	Fine	Ultrafine	Coarse	Fine	Ultrafine
Collected mass (mg)						
Week 1	66	101	22	55	88	23
Week 2	74	142	35	56	117	33

Recovery (%)						
Week 1	54	41	87	70	66	88
Week 2	56	70	68	80	61	101

Ionic components (μg/g)						
Ca^2+^	26,779	3,375	4,464	25,200	3,165	2,796
Mg^2+^	2,879	861	1,902	3,076	1,014	1,918
K^+^	10,051	1,526	2,687	10,840	1,947	2,711
NH_4_^+^	13,819	117,687	153,979	12,975	123,407	157,075
NO_3_^−^	89,121	7,719	8,078	90,681	7,305	8,751
SO_4_^2−^	54,982	372,394	465,643	64,916	411,206	511,638

Carbon species						
Total/PM (%)	19.0	22.3	26.3	20.4	20.2	24.4
Organic (μg/g)	185,455	206,408	261,073	203,073	192,198	243,673
Elemental (μg/g)	4,858	17,038	1,740	508	9,353	0

Endotoxin (EU/g)	253	7	2	102	1	1

**Table 2:** Inorganic elemental constituents of coarse, fine, and ultrafine fractions of PM collected at NR and FR sites (μg/g of PM).

	Al	Ba	Ca	Cd	Cr	Cu	Fe	K	Mg	Mn	Ni	Pb	SO_4_	Sb	Si	Sn	Sr	Ti	V	Zn
NR																				
Coarse	12,000^a^	1,400^c^	22,000	14^a^	48^b^	790^c^	19,000^b^	11,000	6,500	510^a^	33	59^b^	57,000	110^c^	32,000^a^	33	110	970	61	990^c^
Fine	3,600	340^c^	3,600	11	18^a^	250^c^	5,500^b^	3,100	1,400	130	23	67	370,000	54^c^	9,200	76	34	170^c^	42^a^	520^a^
Ultrafine	1,900	10^c^	4,500^b^	18	13	150^a^	1,700^a^	5,900	900	100	30	69	440,000	47	340^c^	21	23^a^	31^b^	54	920
FR																				
Coarse	9,000	540	20,000	11	31	320	10,000	9,900	6,000	390	27	39	63,000	41	24,000	32	100	960	63	400
Fine	3,700	120	3,200	10	14	100	3,000	3,600	1,600	110	23	75	440,000	24	8,100	131^b^	30	74	33	400
Ultrafine	1,800	3	2,600	16	11	110	1,300	5,500	800	90	32	87^a^	450,000	42	170	44^c^	16	20	58	980

NR PM as a percentage of corresponding FR PM:

a 130–150%;

b 150–200%;

c > 200%.

**Table 3:** Hemodynamics at the end of the control period before the ischemia reperfusion.

	Coronary flow rate (mL/min)	LVDP (cm H_2_O)	+dP/d*t*_max_ (mm Hg/sec)	−dP/dt_min_ (mm Hg/sec)	Heart rate (bpm)
NR					
Coarse	2.9 ± 0.6	131 ± 12	4,387 ± 306	−4,267 ± 571	380 ± 41
Fine	2.0 ± 0.7*	119 ± 15	4,093 ± 748	−3,846 ± 1,374	333 ± 42
Ultrafine	1.7 ± 0.7*	144 ± 11	4,663 ± 422	−4,327 ± 305	378 ± 22
FR					
Coarse	3.2 ± 1.5	141 ± 10	4,933 ± 394	−5,017 ± 481	416 ± 10
Fine	3.0 ± 1.4	151 ± 25	4,517 ± 955	−3,223 ± 339	358 ± 29
Ultrafine	2.5 ± 0.7	150 ± 9	4,870 ± 125	−4,129 ± 419	389 ± 18
Saline	3.9 ± 1.4	113 ± 4	3,877 ± 295	−3,045 ± 381	412 ± 17

bpm, beats per minute. PM-exposed animals aspirated 100 μg PM.

* *p* < 0.05 compared with the saline group.
